# Household Hints and Recipes

**Published:** 1873-04

**Authors:** 


### Household Hints & Recipes.

DIABETES.—Dr. O. SCHULTZER, of Dorpat, claims great success in the treatment of diabetes, under the free internal use of glycerine and citric acid, with abstinence from starchy food

---

"SCRATCHES."—To cure scratches on horses, wash the legs with warm, strong soap-suds, and then with beef brine. It is said that two applications will cure the worst case.

---

TO IMPROVE GILDING.—Mix a gill of water with two ounces of purified nitre, one ounce of alum, one ounce of common salt; lay this over gilt articles with a brush, and the color will be much improved.

---

CAMPHOR IN ERYSIPELAS.—Dr. Delpech recommends an ethereal solution of camphor, composed of equal weights of both, a few drops of which are from time to time put upon the erysipelatous surface; in most cases a rapid cure follows.

---

FOR BURNS.—A correspondent sends us the following recipe: "For burns or scalds apply immediately a soft linen rag or lint saturated with strong spirits of camphor, and keep it wet for an hour. You will be surprised at the relief it affords."

---

CLEANING STOVES.—Stove lustre, when mixed with turpentine and applied in the usual manner, is blacker, more glossy, and more durable than when mixed with any other liquid. The turpentine prevents rust, and when put on an old rusty stove, will make it look as well as new.

CEMENT FOR STOVES.—Wood ashes and salt, equal proportion in bulk of each; reduce to a soft paste with cold water, and fill cracks when the range or stove is cool. The cement will soon become hard.

---

WHEN BEGINNING TO USE GLASSES—Use them as short a time as possible, only in deficient light, or on minute objects, and then change the strain to distant or larger objects. By a judicious attention to these two points, the age of the sight will be retarded many years.—And as reading is one of the luxuries of the age, and one of its most delightful pastimes and amusements, we cannot be too careful of the eyesight, and should study how we may best husband its powers.

---

CELERY AS A NERVINE.—A correspondent of the *Practical Farmer* says: "I have known many men, and women too, who, from various causes, had become so much affected with nervousness, that when they stretched out their hands they shook like aspen leaves on windy days; and by a daily, moderate use of the blanched foot-stalks of the celery leaves as a salad, they became as strong and steady in limbs as other people. I have known others so very nervous that the least annoyance put them in a state of agitation, and they were in almost constant perplexity and fear, who were effectually cured by a daily moderate use of blanched celery as a salad at mealtimes. I have known others cured by using celery for palpitation of the heart.

---

REMEDY FOR CORNS.—When a young man, says a correspondent of the *Country Gentleman*, I used to be very much annoyed by those painful excrescences called "corns," on the toes and feet, until I was told of a very simple but effectual remedy for them by some person. It was to bathe the feet in tepid water, to soften them; then pare them off very closely with a sharp knife; then rub on well green peach-tree leaves; when, after continuing the rubbing once or twice a day, the corns will entirely disappear, and not return, without the cause which first occasioned them. I have often tried the remedy, and never found it to fail yet. It seems to be the prussic acid in the peach-tree leaves that takes them away.

A good corn salve could no doubt be made for winter use, by bruising the peach leaves when green, then boil them in water until the strength is extracted; then take out the leaves, strain the water off the sediment, and add a sufficient quantity of resin, beeswax, tallow, and lard oil to make it soft enough, and simmer down, without burning, until the water is evaporated. Soften and pare the corn, as before directed, spread it on a small piece of cambric or linen, and apply, putting on the sock or stocking carefully, so as not to rub it off. Keep applying until the corn disappears.

---

DAMP-PROOF ADHESIVE LABELS FOR BOTTLES.—Macerate five parts of good glue, in eighteen to twenty parts of water for a day, and to the liquid add nine parts of sugar-candy and three parts of gum arabic. The mixture can be brushed upon paper while lukewarm; it keeps well, does not stick together, and when moistened, adheres firmly to bottles. For the labels to be used in places subject to damp, it is well to prepare a paste of good flour and glue, to which linseed oil, varnish, and turpentine have been added, in the proportion of half an ounce each to the pound. Labels prepared in the latter way do not fall off in damp places.

---

TO REMOVE TAR, TURPENTINE, ETC.—A correspondent of the *Medical and Surgical Reporter* has accidentally discovered that a mixture of the pulverized extract of liquorice with oil of aniseed will dissolve and remove Venice turpentine, tar, pitch, or any sticky substance that resists soap and water, from glass, porcelain, or the human body. It is entirely harmless to the skin, and yet will remove these substances as promptly and as thoroughly as soap and water will remove common dirt.

For cleansing tar or pitch from the skin, make a mixture about the consistency of thick cream, and rub on thoroughly with the hand; then follow with a piece of good soap, a sponge, and warm, soft water.

---

VAPOR OF AMMONIA IN WHOOPING COUGH.—Mr. John Grantham states, in the *British Medical Journal*, that in cases of whooping-cough in the last stages (that is, after the third week) he has had one ounce of the strongest liquid ammonia put into a gallon of boiling water in an open pan, and the steam kept up by means of half a brick made red-hot throughout and put into the boiling water containing the ammonia, the pan being placed in the centre of a room, into which the patients were brought as the ammoniated steam was passing off. "This method was used in the evening, just before bed-time; and it has been so efficacious," he says, "in abating the spasmodic attack, and after three or four days terminating the malady, that I cannot overestimate the great value of this mode of inhaling the ammonia, as a therapeutic agent in tranquilizing the nervous system in whooping-cough."

Borax in Colds.—Dr. J. W. Corson, of Orange, N. J., has published some interesting notes on the use of borax and nitrate of potass. in aiding the voice, as sometimes required by singers, clergymen, and public speakers. The value of nitre, or sal prunelle, has long been recognized, and if it is increased by the aid of borax the fact is worth knowing. Dr. Corson sums up the result of his inquiries as follows :—

1. That in sudden hoarseness or loss of voice in public speakers or singers, from "colds," relief for an hour or so, as by magic, may be often obtained by slowly dissolving and partially swallowing a lump of borax the size of a garden-pea, or about three or four grains, held in the mouth for ten minutes before speaking or singing. This produces a profuse secretion of saliva, or "watering" of the mouth and throat. It probably restores the voice or *tone* to the dried vocal cords, just as "wetting" brings back the missing notes to a flute when it is too dry.

2. Such "colds" may be frequently "broken up" at the very commencement; and this restorative action of the borax to the voice may be materially aided by promptly taking, the evening previous to a public effort, dissolved in a glass of sweetened water, a piece of the nitrate of potass. or "saltpetre," a little larger than a garden-pea, or about five grains, on going to bed, and covering with an extra blanket. The patient should keep warm next day. This both moistens the dry throat and further relieves the symptoms of "cold" and slight blood-poisoning from suppressed perspiration, by reopening the millions of pores of the skin more or less closed by cold.

3. These remedies have the three recommendations of being easy to obtain, convenient to carry in traveling, and perfectly harmless.

4. They are nearly or quite useless in the actual cure of long-continued chronic disease of the throat, or acute inflammation, or "tonsillitis," both of which require other appropriate treatment.

